# Bilateral Breast Involvement by Actinomyces neuii in an Immunocompetent Male Patient: An Exceptional Case

**DOI:** 10.7759/cureus.91526

**Published:** 2025-09-03

**Authors:** Pushyami Satya Bandi, Adiraj Singh, Rami Al-Handola, Srujan Edupuganti

**Affiliations:** 1 Internal Medicine and Pediatrics, Hurley Medical Center, Michigan State University, Flint, USA; 2 Internal Medicine, Hurley Medical Center, Michigan State University, Flint, USA

**Keywords:** abscess, actinomyces neuii, actinomycosis, breast, male

## Abstract

*Actinomyces neuii* is a gram-positive, non-branching, rod-shaped bacterium that rarely causes infection in humans. To our knowledge, we report the first case of primary bilateral breast actinomycosis caused by *A. neuii* in an immunocompetent male patient. A 40-year-old man presented with chronic bilateral green nipple discharge. Despite a prior left breast abscess drainage and six weeks of oral penicillin V, symptoms persisted. Culture of the nipple discharge identified *A. neuii*. The patient's history included nipple piercings, and a mammogram confirmed the abscess. After failure of penicillin V, he was switched to oral linezolid, which led to resolution of the discharge, though induration remained on follow-up. Recognizing atypical actinomycosis, thorough evaluation, and microbiological confirmation are key to effective treatment and better patient outcomes.

## Introduction

Primary breast actinomycosis is a rare, chronic granulomatous infection most often caused by *Actinomyces israelii* and typically described in female patients, particularly those of reproductive age. It often presents as a subareolar mass, abscess, or draining sinus, mimicking inflammatory carcinoma or chronic mastitis, which complicates diagnosis and delays appropriate therapy [1-3]. Male breast involvement is exceptionally uncommon, with fewer than 32 cases reported since 1893 [1].

*Actinomyces neuii* is an unusual pathogen within the *Actinomyces *genus. It is a catalase-positive, gram-positive, non-filamentous rod capable of aerobic growth [4-6]. These traits distinguish it from more typical anaerobic branching *Actinomyces* species. It is commonly part of the normal flora of the skin and female genitourinary tract [4], but it rarely causes infections in humans. When it does, infections may include infected epidermoid cysts, prosthetic device-related infections, urinary tract infections, or soft tissue abscesses [5,7,8].

Breast abscesses due to *A. neuii* have been described in only a limited number of cases, nearly all involving female patients. Leenstra et al. reported two cases of *A. neuii *breast infections in females treated successfully with prolonged antibiotics [8]. Mahendiran et al. described the only known male patient with breast actinomycosis; however, he was immunocompromised due to HIV infection, and the specific organism was not *A. neuii *[1]. To our knowledge, there are no published cases of bilateral breast abscesses caused by *A. neuii *in an immunocompetent male.

We present a case of primary bilateral breast actinomycosis caused by *A. neuii *in a 40-year-old immunocompetent male with a history of nipple piercings. This case highlights the importance of careful clinical assessment and microbiological confirmation in the evaluation of chronic or atypical breast infections.

## Case presentation

**Figure 1:**
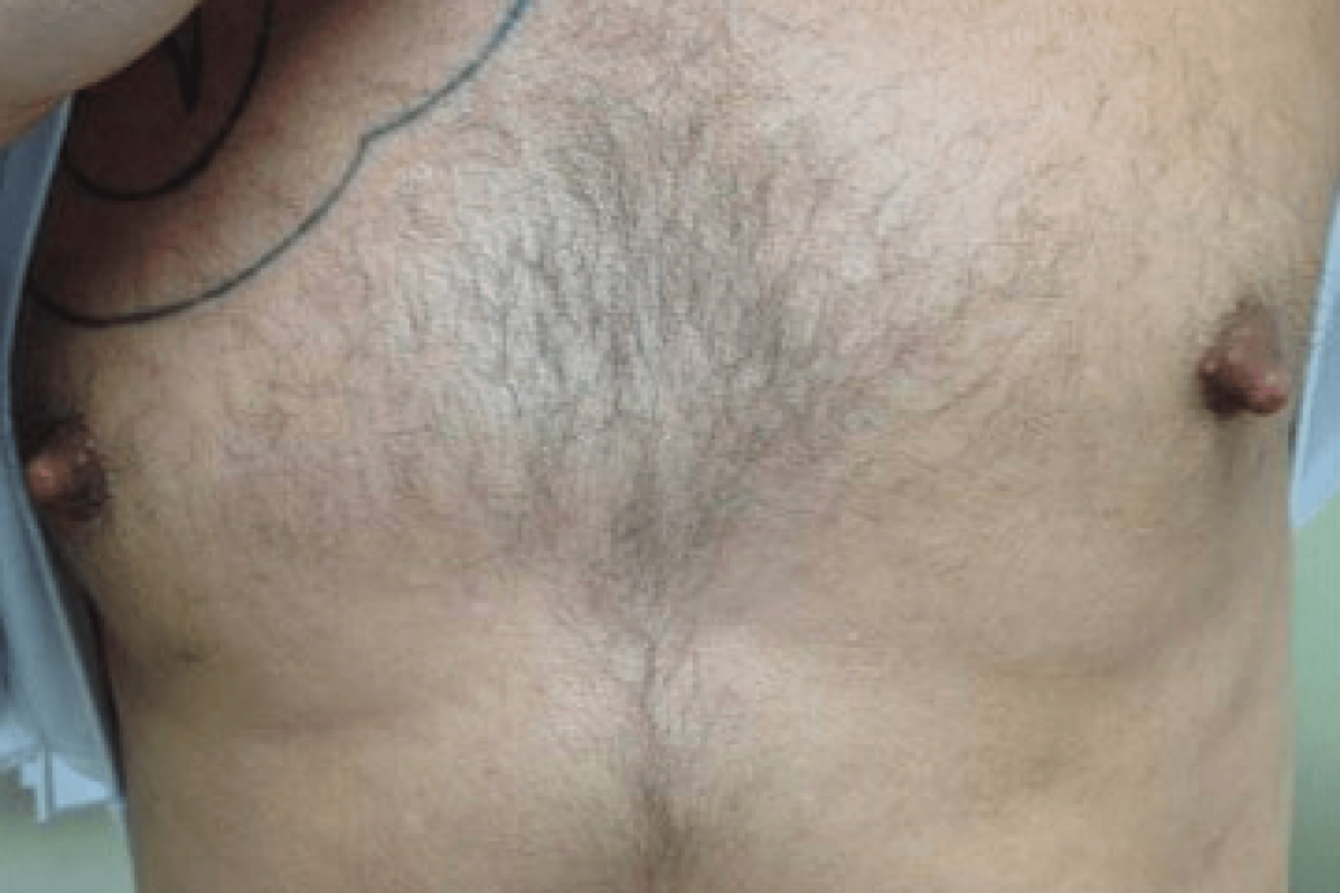
Initial appearance of the patient with bilateral breast swelling.

A 40-year-old male with a history of chronic hepatitis C, depression, anxiety, and substance use (alcohol, marijuana, and tobacco) presented to his primary care physician with several months of spontaneous bilateral nipple discharge. The discharge was thick and “lima bean green” in color. He also noted progressive bilateral breast swelling (Figure 1). He denied fever, chills, weight loss, or trauma. He had previously removed bilateral nipple piercings but had no history of breast surgery or immunodeficiency.

On examination, he was afebrile but mildly tachycardic (103 bpm). The bilateral areolar regions were tender, indurated, and draining purulent material directly from the nipples. No axillary lymphadenopathy was present.

Routine labs and hormone levels were obtained (Table 1). The patient underwent incision and drainage of the left breast, and cultures of the purulent material were sent. He was empirically started on a 10-day course of oral trimethoprim-sulfamethoxazole (TMP-SMX) for presumed methicillin-resistant *Staphylococcus aureus* mastitis. Laboratory evaluation was largely unremarkable, except for an elevated erythrocyte sedimentation rate (46 mm/hour; reference 0-20).

**Table 1:** Laboratory investigations.

Parameter	Patient Value	Reference Range
White blood cell count (WBC)	8.8 × 10⁹/L	4.0-11.0 × 10⁹/L
Hemoglobin	17.2 g/dL	13.5-17.5 g/dL
Platelet count	274 × 10⁹/L	150-400 × 10⁹/L
Erythrocyte sedimentation rate	46 mm/hour	0-20 mm/hour
Prolactin	6.6 ng/mL	2.0-18.0 ng/mL
Estradiol	40 pg/mL	10-40 pg/mL (male)
Progesterone	0.4 ng/mL	<0.5 ng/mL (male)
HIV antibody	Negative	Negative
Blood cultures	No growth	No growth

**Figure 2:**
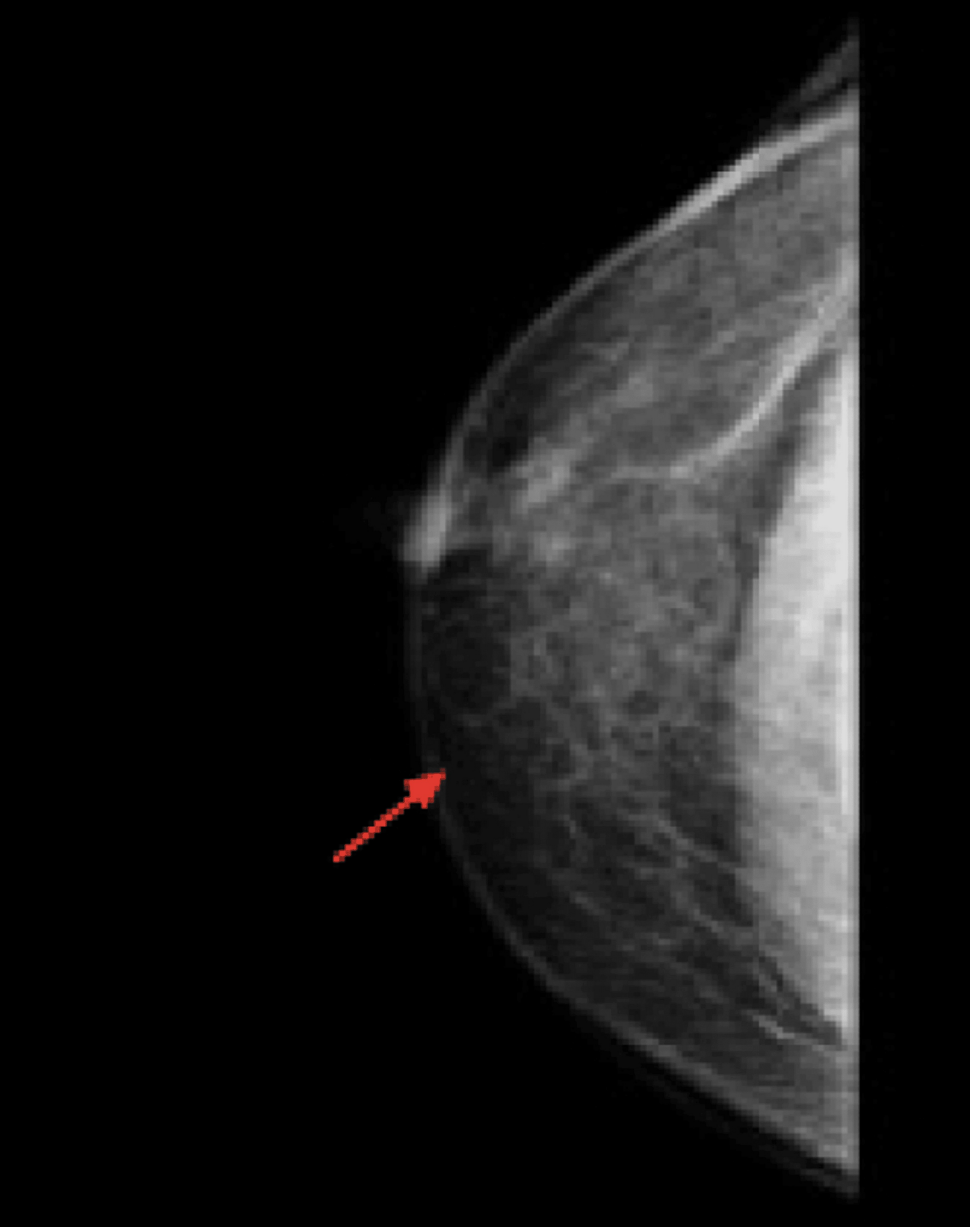
Mammogram (red arrow) showing thick-walled heterogeneous fluid collection contiguous with the overlying skin measuring 0.8 x 0.4 x 1.0 cm in size.

Mammography demonstrated thick-walled, heterogeneous fluid collections contiguous with the overlying skin in both breasts, measuring 0.8 × 0.4 × 1.0 cm (Figure 2), consistent with bilateral abscesses.

Surprisingly, cultures from the drainage grew *Actinomyces neuii*, identified by matrix-assisted laser desorption/ionization time-of-flight (MALDI-TOF) mass spectrometry after two days of anaerobic incubation. Based on this result, he was started on oral penicillin V potassium 500 mg four times daily. Despite nearly one year of compliant therapy, nipple discharge diminished but residual induration persisted.

He was later reassessed in the emergency department for unrelated reasons. Given only partial improvement, he was transitioned to oral linezolid for four weeks, which led to complete resolution of the discharge. However, bilateral areolar thickening remained.

**Figure 3:**
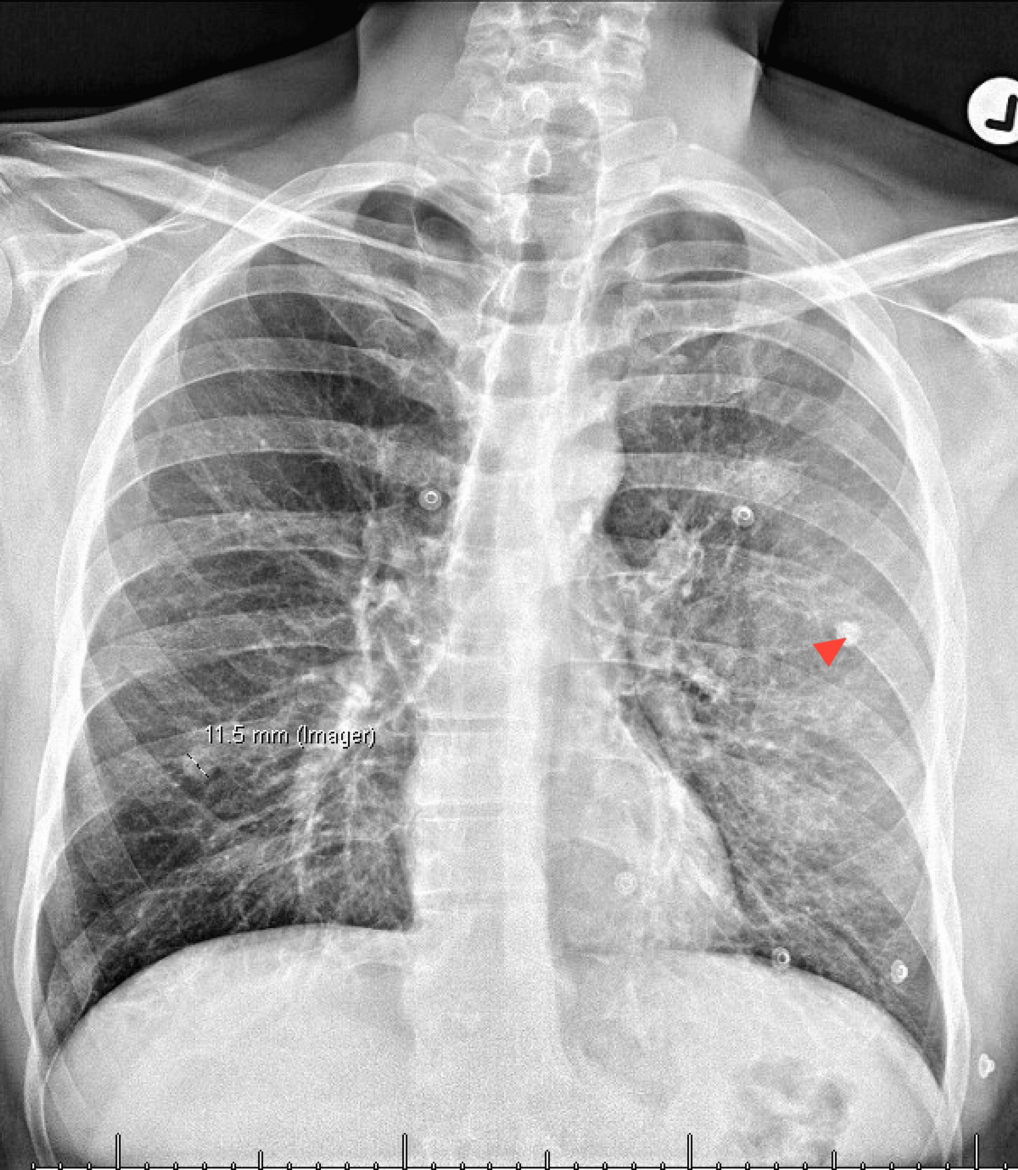
CXR PA view. The red arrowhead shows the calcified granuloma in the left lobe. CXR, chest X-ray; PA, posteroanterior.

**Figure 4:**
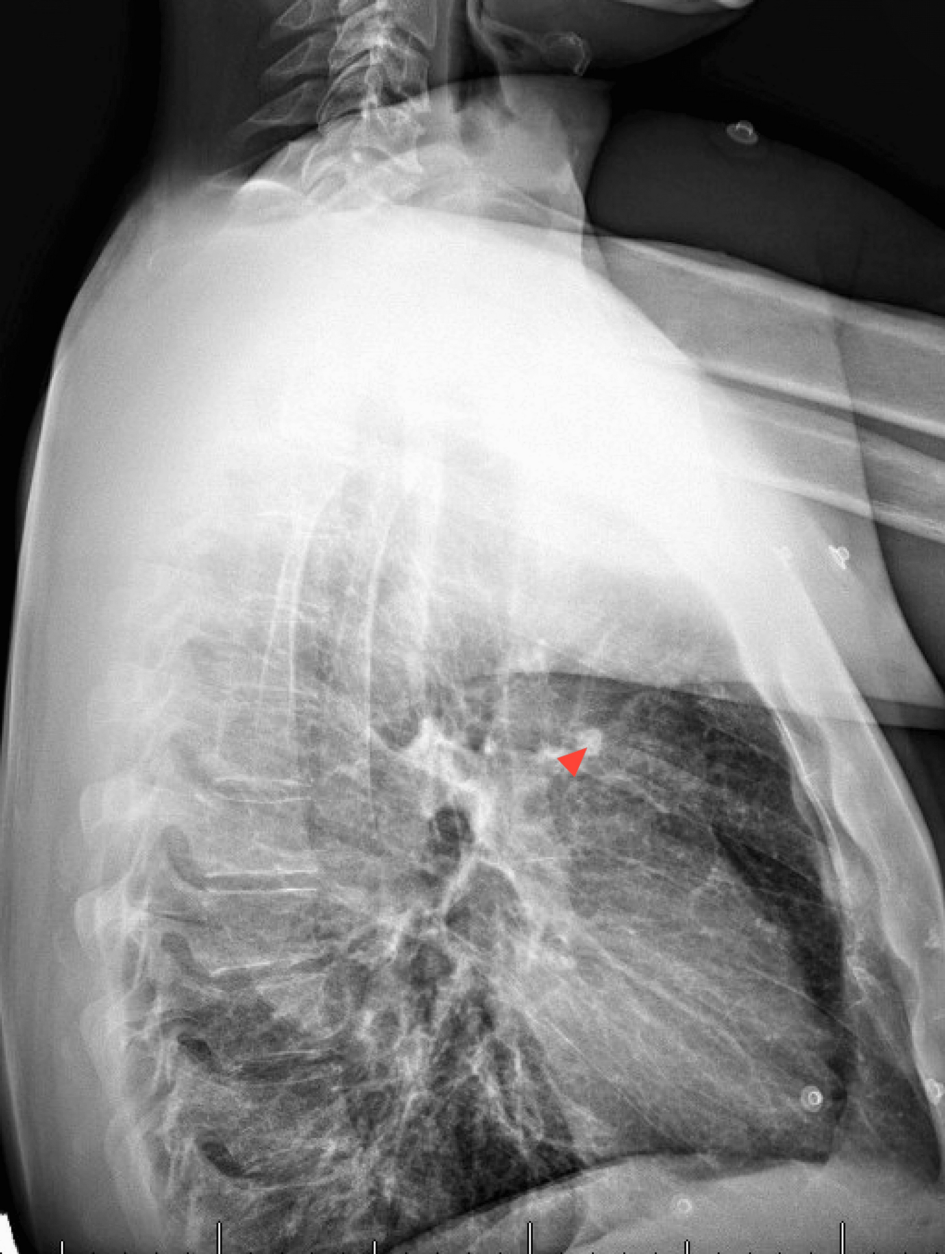
CXR lateral view. The red arrowhead shows a calcified granuloma in the left lobe. CXR, chest X-ray.

Subsequent referral to breast surgery prompted additional imaging. A chest X-ray revealed a calcified granuloma in the left upper lobe (Figures 3, 4). Fine-needle aspiration (FNA) of the breast lesion demonstrated acute inflammation and keratinous debris, consistent with an inflamed epidermoid inclusion cyst coexisting with chronic infection. At three-month follow-up, the patient remained free of nipple discharge, with only mild residual areolar induration.

## Discussion

Anaerobic, gram-positive, branching filamentous bacteria *Actinomyces* spp. are found naturally in the human gastrointestinal tract, urogenital tract, and oropharynx [2,9].

Actinomycosis is a granulomatous infection that is invasive, progressive, and sometimes recurrent [3,9]. The most frequent cause is *A. israelii*, while *A. neuii*, which differs by its aerobic growth and small, non-branching morphology, is a relatively uncommon cause [3-8]. *A. neuii *accounts for roughly 17% of clinical *Actinomyces *isolates [4]. We present what may be the first documented case of primary actinomycosis affecting both breasts caused by *A. neuii *in an otherwise healthy male patient.

*Actinomyces* can infiltrate deeper tissues if the mucous membrane barrier is breached by injury, surgery, or invasive foreign objects. The most likely entry point for the bacteria was the nipple due to his history of bilateral nipple piercings, which led to considerable pain, redness, swelling, and purulent drainage from both breasts over the subsequent weeks. Previous reports of breast actinomycosis due to *A. neuii *are rare, including one male patient with HIV who developed infection following reduction mammoplasty for gynecomastia, which likely facilitated bacterial entry [1], and a 31-year-old female nurse from Iran who developed a comparable infection after undergoing a biopsy of a breast mass [10].

In individuals infected with *A. neuii*, typically we recommend four to six weeks of targeted antibiotics, such as penicillin, clindamycin, or linezolid [3-7,10]. This treatment duration is significantly longer than the standard 7- to 10-day courses we usually prescribe for skin and subcutaneous infections, highlighting the necessity of obtaining cultures whenever feasible in patients presenting with a breast abscess. This case highlights how culture-driven therapy is essential when breast abscesses persist despite standard short antibiotic courses. Surgical intervention is an option for those who do not respond to medical treatment or have recurring infections. The outlook is generally positive. Our case demonstrates persistence and presents an intriguing diagnostic challenge, likely raising numerous questions regarding the etiopathogenesis and management of *A. neuii*.

## Conclusions

In conclusion, this case of primary actinomycosis affecting both breasts caused by *A. neuii *in a healthy male highlights the potential for this organism to cause significant pathology, particularly when barriers such as prior nipple piercings are compromised. This unusual presentation underscores the importance of thorough clinical evaluation and microbiological confirmation through cultures to guide prolonged antibiotic therapy, with surgical intervention reserved for refractory cases. Our patient remained symptom-free three months after completion of therapy, demonstrating the durability of culture-driven treatment. While a single case cannot define practice, these findings suggest the need for larger case series or registries to better understand risk factors, such as nipple piercings, and to optimize treatment duration and strategies for atypical actinomycosis presentations.
